# Opioid-free anesthesia reduces the severity of acute postoperative motion-induced pain and patient-controlled epidural analgesia-related adverse events in lung surgery: randomized clinical trial

**DOI:** 10.3389/fmed.2023.1243311

**Published:** 2023-11-06

**Authors:** Shuwei Wang, Yi Li, Chao Liang, Xiaodan Han, Jiaxing Wang, Changhong Miao

**Affiliations:** Department of Anesthesia, Zhongshan Hospital, Fudan University, Shanghai, China

**Keywords:** video-assisted lung surgery, opioid-free anesthesia, acute postoperative pain, PCEA-related adverse event, opioid-sparing anesthesia

## Abstract

**Background:**

Opioids have been used as pain relievers for thousands of years. However, they may also cause undesirable side effects. We therefore performed this study to compare the effect of opioid-free anesthesia (OFA) versus opioid-sparing anesthesia (OSA) on postoperative pain and patient-controlled epidural analgesia (PCEA)-related events.

**Methods:**

This is a single center randomized clinical trial that was recruited patients aged from 18 to 70 years who received video-assisted lung surgery between October 2021 and February 2022. Participants were 1:1 randomly assigned to OFA or OSA. Patients in the OFA group received propofol, rocuronium, esmolol, lidocaine, and magnesium sulfate intravenously with epidural ropivacaine. Patients in the OSA group received propofol, rocuronium, remifentanil, and sufentanil intravenously with epidural hydromorphone and ropivacaine.

**Results:**

A total number of 124 patients were randomly allocated to the OFA or OSA group. In the OFA group, the severity of pain during coughs on the first postoperative days (PODs; VAS score 1.88 ± 0.88 vs. 2.16 ± 1.1, *p* = 0.044) was significantly lower than that in the OSA group. The total ratio of PCEA-related adverse events in the OFA group [11 (19.6%) vs. 26 (47.3%), *p* = 0.003] was significantly lower than in the OSA group.

**Conclusion:**

OFA in patients who received video-assisted lung surgery led to lower severity of acute postoperative motion-induced pain and fewer PCEA-related adverse events on the first POD than in the patients in the OSA group.

**Clinical trial registration:**

clinicaltrials.gov, identifier (NCT05063396).

## Introduction

Opioids are widely used in pain relieving but may also cause undesirable side effects, including respiratory depression, sedation, nausea, and vomiting (1). Recent studies have shown that opioids may induce immunosuppression, leading to an increased risk of infection (2–4). Furthermore, opioid addiction caused by abuse of prescribed opioids has created a number of health and societal problems, collectively named the ‘opioid crisis’, associated with high opioid-related mortality and morbidity (5, 6).

Due to the location of the incision and the necessity of indwelling chest tubes, thoracic lung surgery was considered as one of the most painful surgical operations demanding highest amounts of opioids perioperatively (7). Opioid-based anesthesia follows the traditional practice of utilizing opioids as a central component of pain management during surgery, while this approach had several potential drawbacks such as respiratory depression and the risk of opioid dependence. The opioid-sparing approach has been recommended by guidelines for enhanced recovery after lung surgery (8). Opioid-sparing anesthesia seeks to minimize opioid use while still employing them to some degree, primarily aiming to reduce the total opioid dose required for pain control postoperatively. In this method, opioids and nonopioid medications are administered together to achieve adequate pain management while minimizing the side effects. Reduced opioid usage during surgery may have the undesired increasing postoperative pain and opioid consumption (9). Nevertheless, the routine use of intraoperative opioids could be entirely avoided by the administration of hypnotics, loco-regional anesthetics, anti-inflammatory drugs, α-2 agonists, and ketamine, i.e., opioid-free anesthesia (OFA) (10). OFA involves the complete avoidance of opioids, opting for alternative medications and techniques to manage pain during surgery, a practice often employed to mitigate opioid-related risks and side effects. Although OFA has been examined in several studies, including bariatric (11), gynecologic (12–15), urologic (13, 14), plastic (13, 14), thoracic (16), and orthopedic (17, 18) surgical trials, no high-quality prospective investigations have been conducted. The safety and feasibility of certain OFA protocols have also been questioned (13). Moreover, previous studies yielded contradictory results concerning the impact of OFA on postoperative pain relief (12, 19, 20). In laparoscopic sleeve gastrectomy, multimodal and intraoperative OFA showed similar pain scores (21). Moreover, substituting opioids with esketamine effectively decreases the occurrence of mild chronic postoperative pain and minimizes side effects in patients undergoing video-assisted thoracoscopic surgery (22). Hence, there is an urgent need for the development of a standardized and robust OFA protocol to facilitate clinical decision making in lung surgery.

This randomized clinical trial aimed to compare the effectiveness of the OFA protocol with the OSA approach in controlling acute postoperative pain in lung surgery patients and the incidence of patient-controlled epidural analgesia (PCEA)-related adverse events.

## Methods

### Study design, setting, and participants

This prospective, randomized parallel-group, single-center, superiority trial was conducted at Zhongshan Hospital affiliated to Fudan University, Shanghai, China, between October 10th 2021 and February 15th 2022. Ethical approval was granted by the local Institutional Review Board of Zhongshan Hospital, affiliated to Fudan University (IRB# B2021-268, approved 22 April, 2021). Written informed consent was obtained from all subjects participating in the trial, which was registered prior to patient enrollment at clinicaltrials.gov (NCT05063396). This manuscript adheres to the Consolidated Standards of Reporting Trials (CONSORT) checklist.

The following inclusion criteria were implemented: (1) age between 18 and 70 years; (2) body mass index [BMI, calculated by weight (kg)/height (m)^2^] between 18 and 30; (3) classified by the American Society of Anaesthesia (ASA) physical status as class I or II; and (4) patients that were planned for selective video-assisted lung surgery. Patients with preoperative chronic use of opioids or NSAIDs, allergy to any drugs included in the protocol, abnormal coagulation, liver or renal insufficiency, psychiatric or neurological disease, cardiac insufficiency or any type of arrhythmia (excluding I° atrioventricular block with a heart rate > 50 bpm), bradycardia (HR ≤ 50 bpm), and patients converted to open surgery or subjected to unexpected drastic blood pressure fluctuations (including newly onset arrhythmia or sudden intraoperative hemorrhage) were excluded from this study.

### Randomization and blinding

After enrolment, patients were 1:1 randomized into two groups by computer-generated random numbers: OFA group and OSA group. The patients, surgical team, post-anesthesia care unit (PACU), acute pain service (APS), and surgical ward nurses were blinded to the treatment allocation. The anesthesiologist or the principal investigator was the only individual aware of patient allocation, who did not participate in the assessment of patients.

### Interventions

At anesthesia induction in the OFA group patients, a bolus of lidocaine (40 mg) and magnesium sulfate (5–10 mg/kg) were co-administered with a target-controlled infusion (TCI) of propofol (3–5 μg/mL), rocuronium (0.6 mg/kg), and esmolol (0.5–1 mg/kg) to obtund the pressor response to intubation in the absence of opioids. All patients in the OFA group received 10 mL of 0.1875% ropivacaine epidural 10 min before incision, and 4–5 mL per hour of 0.1875% epidural ropivacaine was infused during the operation. The maintenance of general anesthesia included the administration of sevoflurane at a minimum alveolar concentration of 0.7–1.0 (titrated to BIS within 40–60), rocuronium (10–20 mg/h), and intravenous lidocaine (1 mg/kg/h, maximum 300 mg during the surgery).

The anesthesia induction in the participants in the OSA group included the administration of TCI of propofol (3–5 μg/mL), TCI of remifentanil (3–5 ng/mL), lidocaine 40 mg (intravenous bolus), and rocuronium 0.6 mg/kg. Sufentanil (10–20 μg) was given intravenously, and epidural hydromorphone (0.3–0.5 mg diluted in 3–5 mL of 0.9% NaCl) was administered 10 min before incision at the anesthesiologist’s discretion. The maintenance of general anesthesia included inhalational sevoflurane at a minimum alveolar concentration of 0.7–1.0 (titrated to BIS within 40–60) and rocuronium (10–20 mg/h). After the resection of the targeted lung tissue, 10 mL of 0.1875% epidural ropivacaine was used, and 4–5 mL of 0.1875% epidural ropivacaine was added per hour afterwards. No patient in the OSA group received intravenous magnesium.

For both the OFA and OSA groups, in the preparation room, an indwelling epidural catheter was preoperatively inserted in all patients at the T6/7 or T7/8 interspace as per the anesthesiologist’s decision, and 3 mL of 2% lidocaine was used to confirm the correct location of the catheter. After admission into the operation room, arterial blood pressure, EKG and pulse oximetry were monitored (Draeger Infinity Omega-S) with bispectral index (BIS). Dexmedetomidine (0.5 μg/kg) was intravenously administered within 10 min before intubation for all patients. If the heart rate of the patient dropped below 45 bpm during the infusion process, the rest of the dexmedetomidine was to be given after the intubation. Video-assisted lung surgery was performed in all patients using trocars positioned at the fourth or fifth intercostal space at the anterior axillary line, the eighth intercostal space at the axillary midline level, and/or around the inferior angle of the scapula. Intraoperative hypertension was treated by increasing the depth of anesthesia or using antihypertensive drugs, such as esmolol and urapidil. Intraoperative hypotension was initially treated with phenylephrine, ephedrine bolus, or norepinephrine (continuous infusion), followed by a rapid infusion of 200 mL of colloid. In both groups, the specific amount of all drugs administered during the operation was documented, and the intraoperative dose changes were adjusted by the anesthesiologist. Paracetamol and parecoxib were administered during the operation. Ramosetron and sugammadex were given before extubation. All patients received PCEA with 0.12% ropivacaine and 0.4 μg/mL sufentanil for the first postoperative 48 h (3 mL/h, 4 mL per bolus, at a minimal interval time of 10 min) and regular oral or intravenous paracetamol and ibuprofen every 12 h.

### Outcomes and measurements

The primary outcomes included the patient-reported VAS pain scores and the PCEA-related adverse events. Specifically, the level of pain was assessed on the first and second PODs at rest and during coughs by staff from APS. Patients were asked to determine their own ‘acceptable’ pain score based on a VAS between 0 and 10. Incidences of PCEA-related adverse events, including nausea, vomiting, pruritus, hypotension (systolic blood pressure < 90 mmHg), dizziness, hypoxemia, need for rescue medication, VAS score ≥ 4, and temporary pump switch-off, were recorded.

Secondary outcomes included PCEA bolus consumption on the first and second PODs, episodes of postoperative pain (VAS ≥ 4) within 48 h at rest, need for rescue intravenous opioids or any type of painkiller other than the regular oral or intravenous paracetamol and ibuprofen, the amounts of intraoperative opioids and vasopressins, hospital length of day (LOS) and overall expenses. Overall expenses were calculated by the hospital information system, including medication, surgery, and perioperative care fees.

### Sample size calculation

The sample size was determined from a preliminary analysis of 60 patients receiving opioid-sparing anesthesia, whose mean pain score on the first POD while coughing was 2.16 ± 1.01. Considering a decrease of 0.5 points with OFA as clinically relevant, a sample size of 64 patients per group was necessary to obtain a statistically significant difference with a power of 80% and a type 1 error of 0.05. The incidence of overall PCEA-related adverse events after video-assisted lung surgery was approximately 47%. Considering that a 50% decrease with OFA is clinically relevant, the minimum number of patients to be included in each group was 61, with a significance level of 0.05 and a power of 0.8.

### Statistical analysis

All analyses were performed using SPSS 21.0 software (IBM, Armonk, NY). Statistical analyses were conducted following the intention-to-treat (ITT) principle. The quantitative data were presented as mean ± standard deviation or median (P25, P75) and analyzed using the unpaired Student’s *t*-test or Mann–Whitney test for significance. Categorical variables were expressed as frequencies and were analyzed using the Chi-square test. Two-sided *p* < 0.05 were considered to indicate statistically significant differences.

## Results

**Figure 1:**
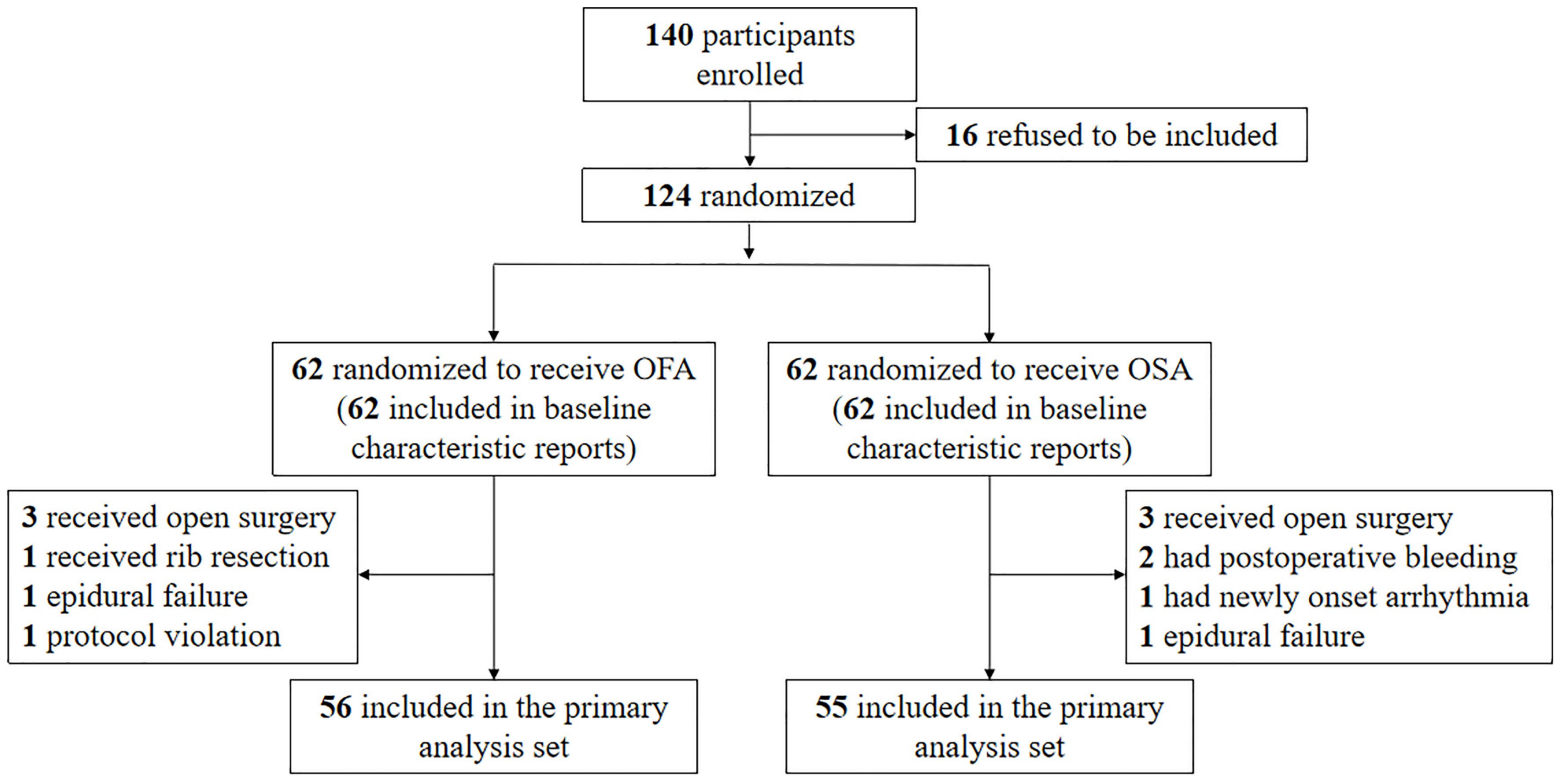
Flow chart.

One hundred and forty patients were initially enrolled, but 16 refused to be included; hence, 124 patients were randomly allocated to the OFA or OSA group. Six patients in the OFA group and seven in the OSA group were excluded (Figure 1). The treatment was completed in one hundred and eleven patients. The patient characteristics between the two groups were comparable, as seen in Table 1. The specific quantities of intraoperative opioids, regional anesthetics, and intraoperative vasopressors are listed in Table 2. The doses of lidocaine, magnesium sulfate, and ropivacaine were significantly higher in the OFA group than in the OSA group (*p* = 0.000). Significantly less ephedrine was administered in the OFA group (4.02 ± 5.21 mg) than in the OSA group (7.98 ± 7.67 mg) (*p =* 0.003). The surgical strategies were also similar between the two groups. The majority of the pathological results in both groups were adenocarcinoma (Table 2).

**Table 1:** Baseline characteristics.

	OFA group *n* = 62	OSA group *n* = 62	*p-*value
Age (years)	50.19 ± 13.01	53.05 ± 12.65	0.218
Male/female	20/42	20/42	1.000
BMI (kg/m^2^)	23.46 ± 3.05	23.15 ± 3.09	0.565
Apfel score (*n*)			0.205
1	9 (14.52%)	11 (17.74%)	
2	17 (27.42%)	8 (12.90%)	
3	30 (48.39%)	33 (53.23%)	
4	6 (9.68%)	10 (16.13%)	
Baseline MBP (mmHg)	77.65 ± 18.72	80.71 ± 19.85	0.262
Baseline HR (bpm)	69.80 ± 13.09	64.22 ± 10.00	0.121
Operative time (h)	1.32 ± 0.68	1.40 ± 0.57	0.469
LOS hospital (days)	5.44 ± 1.46	5.15 ± 1.42	0.264
Overall expenses (¥)	64,617 ± 12,739	66,074 ± 11,999	0.513

BMI, body mass index; LOS hospital, length of hospital stay; Continuous variables are expressed as mean ± SD; Discrete variables are expressed as count (percentage).

**Table 2:** Intraoperative medication, surgical strategies, and pathological diagnosis.

	OFA group *n* = 56	OSA group *n* = 55	*p*-value
**Intraoperative opioid**			
Intravenous sufentanil (mg)	0	12.80 ± 4.05	
epidural hydromorphone (mg)	0	0.39 ± 0.06	
Intravenous remifentanil (ug)	0	108.80 ± 78.60	
**Intraoperative local anesthetics**			
Intravenous 2% lidocaine (mL)	7.84 ± 2.82	2.51 ± 1.15	<0.001
Intravenous magnesium sulfate (g)	0.38 ± 0.14	0	<0.001
Epidural ropivacaine (mL)	18.16 ± 2.65	14.38 ± 1.81	<0.001
**Intraoperative rescue medication**			
Ephedrine (mg)	4.02 ± 5.21	7.98 ± 7.67	0.005
Phenylephrine (mg)	0.25 ± 0.28	0.21 ± 0.30	0.444
Norepinephrine bitartrate (mg)	0.06 ± 0.12	0.03 ± 0.05	0.174
Surgical choices, *n* (%)			0.337
Lobectomy	6 (10.7%)	13 (23.6%)	
Segmentectomy	12 (21.4%)	11 (20.0%)	
Wedge resection	26 (46.4%)	22 (40.0%)	
Combined	12 (21.4%)	9 (16.4%)	
Pathological diagnosis, (*n*)			0.615
Adenocarcinoma	51	50	
Benign or atypical lesions	4	2	
Lymphoma	1	1	
Hamartoma	0	1	
Neuroendocrine tumor	0	1	

Continuous variables are expressed as mean ± SD. Discrete variables are expressed as count (percentage).

### Outcomes

**Figure 2:**
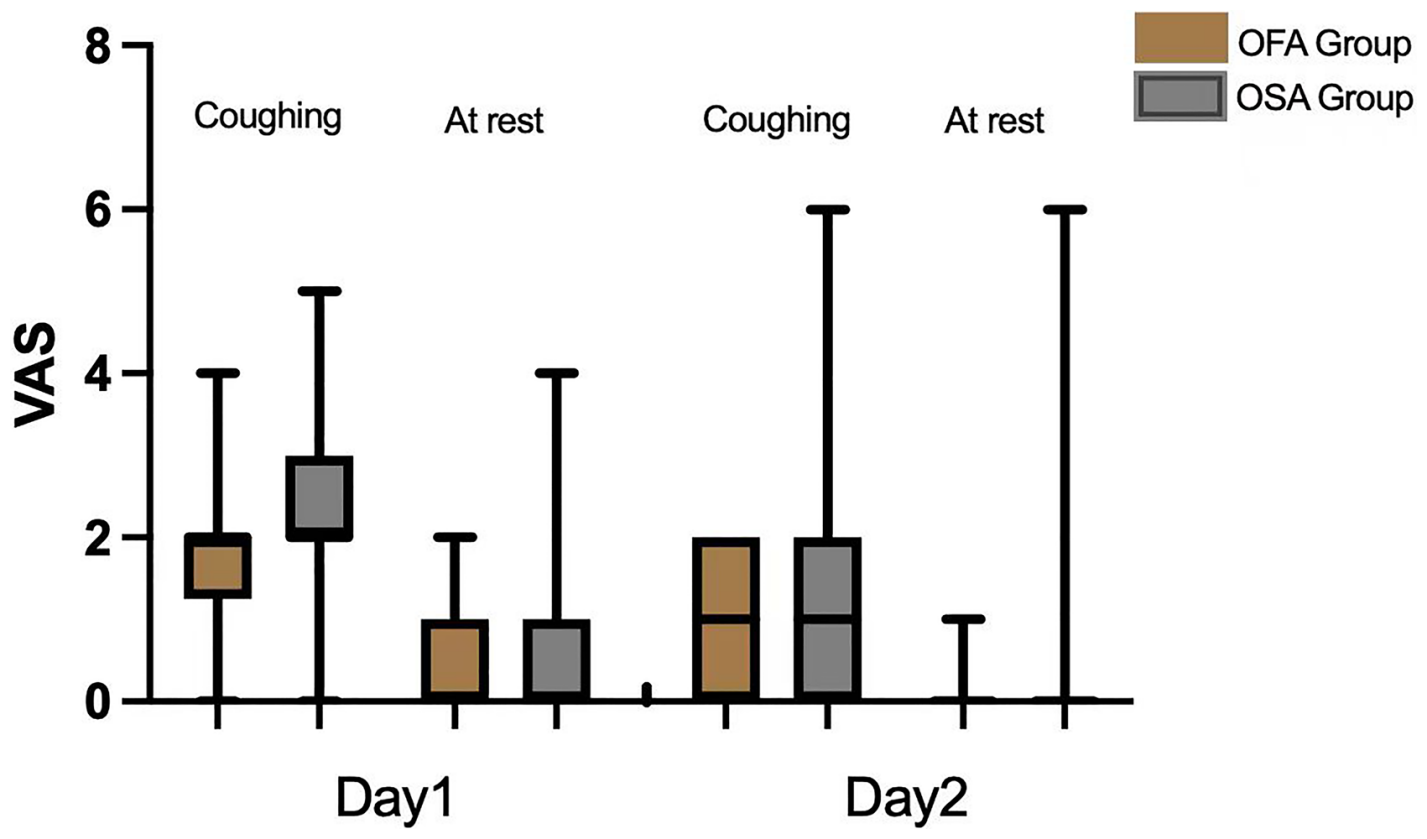
Box plots of postoperative pain scores.

As visible in Figure 2 and Table 3, the mean VAS score on the first POD while coughing was significantly lower in the OFA group [2 (1.25, 2)] than in the OSA group [2 (2, 3)] (*p =* 0.044). No significant difference was found between the OFA and OSA pain scores on the first POD at rest and on the second POD, both at rest and during coughing (all *p* > 0.05). The postoperative PCEA bolus consumptions were also similar in the two groups on the first two PODs (all *p* > 0.05).

**Table 3:** Postoperative pain score and PCEA consumption.

	OFA group *n* = 56	OSA group *n* = 55	*p-*value
**Postoperative VAS score**			
Day 1 at rest	0 (0, 1)	0 (0, 1)	0.966
Day 1 coughing	2 (1.25, 2)	2 (2, 3)	0.044
Day 2 at rest	0 (0, 0)	0 (0, 0)	0.616
Day 2 coughing	1 (0, 2)	1 (0, 1)	0.204
**Postoperative PCEA bolus consumption (time)**			
Day 1	1 (1, 2)	1 (0, 2)	0.724
Day 2	1.5 (1, 3)	1.5 (1, 3)	0.851

Adverse events					
Symptoms related to opioids	Day 1	Day 2	Day 1	Day 2	
PONV (*n*)	2	1	4	3	0.203
Dizziness (*n*)	0	2	2	2	0.438
Pruritus (*n*)	0	0	5	1	0.013
Hypotension (*n*)	0	1	0	0	1.000
Rescue medication (*n*)	1	0	1	0	1.000
VAS score ≥ 4 (*n*)	1	0	3	1	0.206
Temporary pump switch-off (*n*)	2	1	2	2	0.716
Total adverse events	11 (19.64%)		26 (47.27%)		0.003

VAS, visual analogue pain scale; PCEA, patient-controlled epidural analgesia; PONV, postoperative nausea and vomiting.

### Adverse events

Three patients in the OFA group reported postoperative nausea and vomiting (PONV), among whom two decided to temporarily switch off the PCEA after balancing the severity of their PONV and acute pain. Bradycardia was observed in 5 patients during the infusion of anesthesia. There were no reports of hypotension or hypertension during this period. And no cases of bradycardia were documented in the post-anesthesia care unit (PACU), and the nurses in the PACU did not report any occurrences of hypotension or hypertension either. In the OSA group, seven patients experienced PONV, and four switched off PCEA. This observation may be attributed to the utilization of PCEA, insufficient food intake and fluid infusion, as well as heightened perspiration during the procedure. The numbers of temporary pump switch-off event was not significantly different between the two groups. After PONV relief was achieved, PCEA was continued for all these patients. Hypotension was reported by one patient in the OFA group, whose PCEA was switched off temporarily and continued afterwards. Patient-reported VAS ≥ 4 at rest was obtained in one patient in the OFA group, who asked for rescue medication, whereas four patients in the OSA group reported VAS ≥ 4 at rest, among whom only one asked for rescue medication. Overall, the ratio of PCEA-related adverse events in the OFA group (*n* = 11) was significantly lower than that in the OSA group (*n* = 26; *p* = 0.003). PCEA-related adverse events in the OFA and OSA groups is presented in Table 3.

## Discussion

The present study indicated that compared with OSA, the OFA protocol could enhance the acute postoperative pain relief on the first POD during coughing and significantly reduce the PCEA-related adverse events in patients who receive video-assisted lung surgery.

In this study, the severity of pain on the first POD while coughing in the OFA group was significantly lower than that in the OSA group. This could be explained by the avoidance of intraoperative opioids in the OFA group, since opioids (especially short-acting types such as remifentanil) are well-known to trigger hyperalgesia in a dose-related manner (23). The severity of pain on the first POD at rest and on the second POD, as well as the PCEA bolus consumption, were similar in the two groups. The lack of difference may be explained by the relatively low severity of pain at these time points (median 0 at rest on both first and second PODs, median 1 on the second POD while coughing) and the very few times of PCEA bolus (median 1 time on the first POD and median 1.5 times on the second POD) in both groups. These results are consistent with previous literature evidence that OFA effectively relieved postoperative pain (11, 16, 24, 25).

The amounts of intravenous lidocaine, magnesium sulfate, and epidural ropicaine used in OFA group were significantly more than those in OSA group. This is due to the intrinsic difference between the OFA and OSA protocols. However, such difference may not lead to the differences in the main outcomes or adverse events because there was a time interval of approximately 14–18 h between the surgery and the follow-up on the first POD. Considering the pharmacokinetics of these drugs, the decrease in pain in OFA group was not likely caused by the extra intravenous and epidural local anesthetics during surgery. Meanwhile, consistent with the findings reported by Maeva (25), a significantly higher amount of ephedrine was administered in the OSA group, whereas the amounts of phenylephrine and norepinephrine in the two groups were comparable. In the OFA group, without opioids to suppress the sympathetic system, it is rational to choose other vasopressins over ephedrine when the blood pressure is low and the heart rate is relatively high. In both groups, no severe bradycardia was observed because less dexmedetomidine was used in our protocol compared to that used in the POFA trial (13, 26). Five patients experienced a heart rate ≤ 50 bpm, but none of them reported any related symptoms during the use of dexmedetomidine before intubation. During the insertion of the double-lumen tube, an elevation in the heart rate and blood pressure was observed in the majority of patients in the OFA group, which could be controlled by the use of esmolol at a reasonable level for a short period of time (heart rate < 100 bpm, invasive blood pressure within 25% elevation). However, if the double-lumen tube was inserted to the wrong side or more time was needed due to a difficult airway, additional esmolol and propofol were needed to blunt this extra stimulus, as observed in two patients in the OFA group. Although ketamine and clonidine were included in other OFA protocols to suppress intubation stimulus, only local anesthetics and esmolol were used in our protocol because ketamine could possibly affect cognitive deficits (27) and clonidine might interfere with hemodynamics (28).

It has been shown that vomiting is the first adverse event most patients wish to avoid, ahead of postoperative pain and all other outcomes (29). In this study, PONV was observed in three patients in the OFA group and in seven patients in the OSA group, six of whom accounted for 75% of the temporary pump switch-off events. Consistent with previous findings, the avoidance of intraoperative opioids could reduce the episodes of PONV (30); however, PONV still occurred in 5.3% of the patients in the OFA group due to use of postoperative opioids in the PCEA regimen. All six patients reporting pruritus were in the OSA group, which was presumably induced by the epidural hydromorphone. Hence, the OSA protocol could potentially be optimized in the future to avoid pruritus. Only one patient-reported VAS ≥ 4 (the threshold between moderate and mild pain) in the OFA group compared to four in the OSA group, which reinforced our finding that the OFA protocol could indeed relieve postoperative pain. Although the number of PCEA-related adverse events was significantly lower in the OFA group, no benefits concerning hospital LOS and overall expenses were observed. It should also be noted that no severe OFA protocol-associated hemodynamic adverse events occurred.

Our study suggests that OFA is a vital component of the enhanced recovery after surgery (ERAS) protocol that aims to optimize perioperative care and promote fast patient recovery (19, 31). By minimizing the use of opioids, OFA creates new options for improving patients’ experience with enhanced pain relief and reduced adverse events without additional expenses or prolonged hospital stays. It can thus be expected that OFA, after further optimization, may provide lower expenses and shortened hospital stays in the future compared with OSA. While OFA represents a the promising frontier in enhancing surgical patient safety (32), it is curcial to acknowledge that, given its advantages and disadvantages, OFA may not universally serve as the primary choice for all surgical patients (33, 34).

Besides the encouraging findings, this trial has several limitations. Firstly, the time course of OFA was limited only to the duration of anesthesia. For this reason, when we withdrew opioids from the PCEA regimen, four out of five patients reported severe motion-induced postoperative pain (VAS > 6), and opioids were used as rescue medication in all four patients. Secondly, the patients’ satisfaction with anesthesia and analgesia was not considered. During the postoperative follow-up period, several patients reported tolerable postoperative pain, but the fear and anxiety when receiving epidural anesthesia were unbearable in the preparation room (considering that the duration of the process ranged from several minutes to over 10 min among individuals). Thirdly, the time from extubation to discharge from PACU was not recorded. Prolonged times and deeper sedation levels in PACU were reported in the OFA group in a previous study (12); however, much higher doses of dexmedetomidine were given compared to those administered in the present study. Finally, we did not include the respiratory infectious complications in this study. Further investigations are required to comprehensively evaluate the efficacy and safety of OFA.

## Conclusion

The present clinical trial shows that OFA could reduce motion-induced postoperative pain and incidence of PCEA-related adverse events in video-assisted lung surgery, without any severe adverse events related to the OFA protocol. Thus, OFA may be recommended in video-assisted lung surgery for better pain management and safety. Considering the lack of a recognized and standardized procedure-specific OFA protocol, our results may serve as a foundation for further research in this area and contribute to the future development of universally accepted unified OFA guidelines.

## Data availability statement

The original contributions presented in the study are included in the article/supplementary material, further inquiries can be directed to the corresponding author.

## Ethics statement

The studies involving humans were approved by ethical approval was granted by the local Institutional Review Board of Zhongshan Hospital, affiliated to Fudan University (IRB# B2021-268, approved 22 April, 2021). The studies were conducted in accordance with the local legislation and institutional requirements. The participants provided their written informed consent to participate in this study.

## Author contributions

SW, JW, and CM conceived and coordinated the study. SW, YL, CL, and XH performed, collected, and analyzed the data. SW and CM wrote and revised the manuscript. All authors contributed to the article and approved the submitted version.
